# The Relationship between Caries-Specific Quality of Life and Generic Wellbeing in a Dutch Pediatric Population

**DOI:** 10.3390/dj7030067

**Published:** 2019-07-01

**Authors:** Helen J. Rogers, Jan H. Vermaire, Fiona Gilchrist, Annemarie A. Schuller

**Affiliations:** 1 School of Clinical Dentistry, University of Sheffield, Sheffield S10 2TA, UK; 2 TNO Child Health, Schipholweg 77-89, 2316 ZL Leiden, The Netherlands; 3 UMCG, Centre for Dentistry and Dental Hygiene, P.O. Box 30001 9700 RB Groningen, The Netherlands

**Keywords:** caries, oral health-related quality of life, preference-based measures

## Abstract

Dental caries has significant negative impacts on the lives of children and young people. Whilst the impacts on children’s oral health-related quality of life (OHRQoL) have been increasingly investigated, the effect on children’s overall wellbeing remains largely unknown. Data were obtained from a survey conducted across four cities in the Netherlands. Children and their parents completed a series of questionnaires, which included Dutch versions of a caries-specific pediatric measure of OHRQoL (CARIES-QC-NL) and a generic pediatric health utility measure (CHU9D-NL). The participating children underwent dental examinations to determine their caries status. A total of 486 11-year-old children participated in the study, of which 184 had caries experience (38%). The mean number of decayed, missing and filled teeth (DMFT) was 0.71. The CARIES-QC-NL was found to have statistically significant correlations with the DMFT and CHU9D-NL. There were no statistically significant correlations between the CHU9D and the clinical variables. The CARIES-QC-NL had acceptable internal consistency and construct validity in this population despite the low prevalence of active caries. A relationship was demonstrated between OHRQoL and generic wellbeing in this population. Despite this, the CHU9D did not show any correlation with the clinical data, which may limit its application in studies of the impact of dental caries.

## 1. Introduction

Dental caries is a prevalent disease amongst children and can cause chronic pain, infection and in some cases may lead to emergency hospitalization [1,2]. A systematic review reported 9% of children worldwide to have untreated dental caries in their primary teeth, highlighting it as a major health problem globally [3,4].

Dental caries has a significant impact on children and their families. Toothache was reported by 18% of 12-year-olds and 15% of 15-year-olds in the UK, with a higher prevalence of up to 30% in younger children from deprived areas [5,6]. In the Netherlands, 12% of 5-year-olds were found to have encountered toothache at least once in their life. For 11-year-olds this percentage was much higher at 21% [3]. Further impacts relating to dental pain have also been widely reported such as time off school, difficulty sleeping and speaking as well as interference with daily activiti4es [7,8,9].

There is a growing body of evidence on the impacts of caries on children’s daily lives and the effect of dental interventions in improving children’s oral health-related quality of life (OHRQoL) [10,11,12]. A number of measures of children’s OHRQoL are available but many have inherent limitations [13]. A key concern is that these measures are not condition-specific and may fail to capture the impacts of caries. Furthermore, they were not developed to assess treatment-related changes and may lack the psychometric properties to do so.

In view of the perceived need for a caries-specific measure of OHRQoL, which is sensitive to change, a measure was developed and validated for clinical use with children [14,15]. The 12-item instrument, known as the Caries Impacts and Experiences Questionnaire for Children (CARIES-QC), seeks children’s assessment of the severity of their caries-related impacts and is appropriate for use with 5–16-year-olds (see Supplementary Material). This unidimensional measure of OHRQoL specific to dental caries has been shown to have good face, content and construct validity, responsiveness and reliability in a UK population [16]. Its validity in other populations has yet to be demonstrated.

Whilst the impact of caries on children’s OHRQoL is being increasingly investigated, the impact of caries on children’s overall health-related quality of life has not been so widely explored. General health-related quality of life (HRQoL) in children is typically reported using generic pediatric utility measures, which can be used across all health conditions. One such measure is the Child Health Utility 9 Dimensions (CHU9D), which was developed for use with children aged 7 to 17 years and has been subsequently translated into seven languages, including Dutch. Children and young people were involved in the development of the CHU9D, to ensure that the items within each dimension were relevant to children, and that the language used was appropriate [17]. A Dutch value set was also gained using values from the adult population, to produce the CHU9D-NL [18]. An advantage of these preference-based measures is their ability to be used to generate the quality-adjustment weight for quality-adjusted life years, known as QALYs. The QALY combines the length and quality of life gained as a result of a healthcare intervention into a single, comparable unit, which can be used by decision-makers to guide resource allocation. Nonetheless, the application of generic preference-based measures to oral conditions such as dental caries remains unclear.

This study aimed to identify the extent to which caries-specific quality of life is related to generic wellbeing, as measured with the CHU9D-NL in Dutch 11-year old children and to validate the CARIES-QC-NL in this population.

## 2. Materials and Methods

Data were collected as part of a wider survey commissioned by the Dutch National Health Care Institute (ZINL). The sample was drawn from a population of 11-year-old children and their parents living in Alphen aan den Rijn, Gouda, Breda and ‘s-Hertogenbosch. A *t*-test-based power calculation showed that a target sample of 460 children was sufficient to determine a statistically significant 30% difference in the number of decayed, missing and filled surfaces (DMFS) between groups (alpha: 0.05, beta: 0.8). Taking into account an anticipated dropout-rate of 20%, a total of 550 children were invited to participate in this study. The study information was sent to parents, with an invitation to provide consent for both their child and themselves to participate. This study was judged by the Central Committee on Research involving Human Subjects (CCMO) not to fall under the Medical Research Involving Human Subjects Act. It met all the requirements of the Personal Data Protection Act (m1501261).

Signed informed consent forms and completed questionnaires were received for a total of 486 children, who were therefore included in this study. Participating children were required to undergo dental examinations, which were carried out using a mirror, probe, 3-in-1 syringe and light source. Numerous dental features were recorded, including the number of decayed, missing and filled surfaces in both the primary and permanent dentitions (dmfs/DMFS), the number of decayed, missing and filled teeth in both the primary and permanent dentitions (dmft/DMFT), as well as gingival health. The examining dentists had undertaken several calibration sessions prior to the examinations. Further detail on the examination procedure is provided in the report of the overall survey findings [3].

Both parents and children were asked to complete a series of questionnaires. Parents received the questionnaires via regular mail, whilst children completed them prior to or following the clinical examination. The questionnaires requested socio-demographic details, alongside information regarding dental anxiety, current oral hygiene practices and satisfaction with dental care. The questionnaires also contained the Dutch translations of the CARIES-QC and CHU9D measures.

### 2.1. CARIES-QC-NL

CARIES-QC is a recently developed caries-specific measure of OHRQoL developed with children with dental caries. CARIES-QC contains 12 items relating to potential impacts of dental caries and one global question, which asks “How much of a problem are your teeth for you?” (Table 3). The items are scored on a 3-point Likert scale (“Not at all”; “A bit” and “A lot”) and scored 0–2 with increasing score indicating increasing severity of the impact (and, therefore, a lower OHRQoL).

The Dutch version of the CARIES-QC (CARIES-QC-NL) was translated by an ISO 17100-certified translation provider, specialized in patient-reported outcome measures (certificate number 2493-TX-0003). The procedure entailed concept elaboration, dual forward translation (including reconciliation), dual back translation (including a review by the CARIES-QC developer), cognitive debriefing by five Dutch native speaking children aged 5–16 years with dental caries and proofreading by a separate professional linguist.

### 2.2. CHU9D-NL

The CHU9D consists of nine dimensions: worried, sad, pain, tired, annoyed, schoolwork/homework, sleep, daily routine, and activities. Each dimension is represented by a single question with five ordinal response options (I don’t feel worried today; I feel a little bit worried today; I feel a bit worried today; I feel quite worried today; I feel very worried today). The recall period is today/last night. The responses can be taken together as a description of the HRQoL of the child; this is termed a health state. There are many different health states defined by the CHU9D descriptive system (due to the different combinations of response options on each of the nine dimensions), and each unique health state has a preference weight associated with it. These preference weights give a utility value (on a 0–1 scale, where 1 is perfect health and 0 is a state equivalent to being dead) which, when combined with the length of life, enables the calculation of the QALY. For the purpose of this study, the Dutch value set was used to calculate utility values. The translation of the Dutch version of CHU9D (CHU9D-NL) is detailed elsewhere [18].

### 2.3. Statistical Analysis

Data were analyzed using SPSS (version 25.0; Armonk, NY, USA) and computation of descriptive statistics were followed by appropriate bivariate analyses.

**Figure 1:**
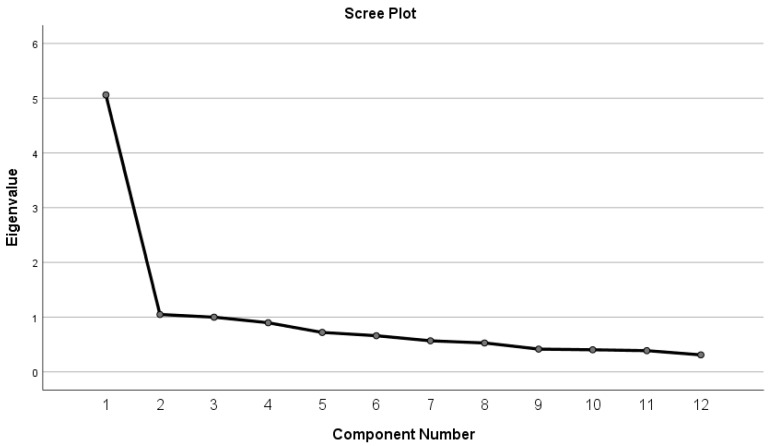
Scree plot demonstrating the elbow following the first component.

The fit and function of the CARIES-QC-NL items were examined using an item response theory Rasch model [19]. In addition, the items were assessed to ensure they were free from differential item functioning (DIF). That is, that they function in the same way between the two populations (UK and Dutch) and between different subgroups. The methods used were the unrestricted or partial credit model suggested by Tennant and Conaghan [20]. The Rasch analysis was undertaken using RUMM2030 (RUMM Laboratory PtyLtd, WA, Australia). Furthermore, the unidimensionality of the CARIES-QC-NL was confirmed at the outset using factor analysis. As seen in the Scree plot (Figure 1), the first component (Eigenvalue 5.056) accounts for the largest percentage of the total variance (42.137%), with each successive factor after this accounting for smaller amounts of the total variance.

Cronbach’s alpha was calculated for CARIES-QC-NL to assess internal consistency in this population. Cronbach’s alpha of > 0.7 is accepted as indicating a homogenous scale [21]. To assess convergent construct validity, it was hypothesized that there would be positive correlations with CHU9D-NL and the global score of CARIES-QC-NL. It was also hypothesized that there would also be positive correlations between the CARIES-QC-NL total score and the clinical data, thereby assessing its concurrent construct validity.

A total of 37 of the 523 participating children omitted to fill out one or more items of the CHU9D-NL or CARIES-QC-NL questionnaires and were, therefore, eliminated from further analysis of the measures. The composition of the participating children did not statistically significantly differ from the remaining group.

## 3. Results

### 3.1. Characteristics of the Sample

The total sample comprised 486 children, of which 184 (38%) had caries experience with International Caries Detection and Assessment System (ICDAS) scores of 4 or higher (caries group) and 302 (62%) children had no caries experience or an ICDAS score of 3 or lower (caries-free group). Both groups had similar proportions of boys and girls (caries group: 52% male (*n* = 95) and 48% female (*n* = 89); caries-free group: 49% male (*n* = 148) and 51% female (*n* = 154). The majority of children in each group were born in the Netherlands, with a small proportion of children who were born elsewhere (4%; *n* = 8 and 5%; *n* = 14, respectively). Most children in each group had mothers who were educated to high school level or higher (caries group: 57% (*n* = 105); caries-free group: 66% (*n* = 199). Further detail is shown in Table 1.

Participants had a mean D_3_MFT of 0.71, with a range of 0–8 teeth and 0–11 surfaces having caries experience. The mean number of carious teeth was 0.36. Further details regarding caries experience are shown in Table 2.

### 3.2. CARIES-QC-NL

A total of 486 participants fully completed the 12 items in CARIES-QC-NL, with 7% (*n* = 37) of participants failing to complete the items. Of those whom completed the CARIES-QC-NL items, 38% (*n* = 184) had caries experience (of which 124 (67%) had untreated decayed teeth) and 62% (*n* = 302) had no caries-experience. The CARIES-QC-NL demonstrated excellent internal consistency (Cronbach’s alpha = 0.9) and further analysis revealed that Cronbach’s alpha would not be improved if any question was removed. All items demonstrated a good fit to the model using the Rasch method with fit residuals in the range ± 2.5, and summary chi-square of *p* = 0.03. There was no DIF identified in the Dutch participants when assessing socioeconomic status and gender. DIF was detected between the original UK participants and the Dutch participants with caries in how they answered the items regarding “food gets stuck in my teeth” and “cried about my teeth”, with UK participants more likely to endorse these items than their Dutch counterparts.

The mean person location for the Dutch participants was −3.59 compared to −1.12 for those in the UK when the items were centered on zero, which confirmed the finding that the Dutch participants reported fewer impacts than their UK counterparts. This was likely to be due to the difference in the number of carious teeth between the groups (mean number of decayed teeth = 0.36 in the Dutch group compared to 6.01 in the UK group).

Table 3 shows the distribution of answers to the CARIES-QC-NL questionnaires in children with and without caries experience, whilst Table 4 shows the overall scores by sub-group. Item scores of “teeth hurt”, “hard to eat some foods” and “food gets stuck in my teeth” were statistically significantly different between both groups, with children with caries experience reporting more frequently having encountered these problems. The item which had the highest proportion (17%; *n* = 88) of participants reporting that impact related to getting food stuck in the holes in their teeth. Regarding the frequency of impacts, the questions regarding feeling “cross” about their teeth and eating on “one side” of their mouth had the highest proportion (2%; *n* = 11 each) of all participants, answering that this affected them “a lot”. Only 2% (*n* = 10) children reported that their teeth had an impact on their schoolwork. In answer to the global question, which asks respondents how much of a problem their teeth are for them, 66% of children (*n* = 321) reported that their teeth did not cause them a problem.

The item related to having pain from their teeth was experienced by 20% (*n* = 38) of children with caries, compared with 11% (*n* = 37) of those without caries. A similar proportion of children with and without caries (36.2%; *n* = 68 and 33.8%; *n* = 103, respectively) reported that their teeth were a problem in answer to the global question.

There were weak but statistically significant (*p* < 0.05) correlations between the CARIES-QC total score, and DMFT (*r* = 0.092) and DMFS (*r* = 0.107). Further statistically significant correlations were found between the total number of missing teeth (r = 0.092) and the total number of filled teeth and surfaces (*r* = 0.113 and *r* = 0.114, respectively). In addition, there was a significant correlation (*p* = 0.01; *r* = 0.279) between the CARIES-QC total score and the global question.

Sub-analysis of the children with a DMFT/DMFS > 0 revealed a significant correlation between the CARIES-QC and the number of filled teeth and surfaces (*r* = 0.153, *p* < 0.05), and between the CARIES-QC and the global question (*r* = −0.362, *p* = < 0.001), which demonstrated slightly stronger correlations when considering only those with caries experience.

### 3.3. CHU9D-NL

The CHU9D-NL was completed by 486 participants and their parents. The mean (range; standard deviation (SD)) utility score for participants was 0.93 (0.36–1.00; 0.08). The parental rating using the CHU9D was slightly higher (0.95; range = 0.15–1.00; SD = 0.09). Overall, a statistically significant negative correlation (Pearson correlation coefficient = −0.115, *p* = 0.011) was found between the child CHU9D utility score and the CARIES-QC total score. This would be expected, as a higher CARIES-QC-NL score indicates increased impacts resulting from caries and a lower CHU9D-NL score indicates an increased impact on generic wellbeing.

No statistically significant correlations were found between the CHU9D and the clinical variables, nor could differences in the CHU9D-NL-derived utility scores could be identified (0.949 in the caries-free group and 0.946 in the caries group). Bivariate correlations between the measures for each group are shown in Table 5.

## 4. Discussion

This study aimed to assess the relationship between OHRQoL and general wellbeing in Dutch schoolchildren and to validate the CARIES-QC-NL in this population. The data reported in this manuscript formed part of a larger epidemiological study assessing oral health behaviors and the children were a representative sample of children from four cities in the Netherlands. The present study demonstrated a statistically significant relationship between general wellbeing and oral health, as determined using the CHU9D-NL and CARIES-QC-NL measures, respectively. Furthermore, a statistically significant association between the clinical data and CARIES-QC-NL provided confirmation of the validity of the Dutch version of this measure.

This was the first time the Dutch version of the CARIES-QC had been used (CARIES-QC-NL). It showed excellent internal consistency, as would be expected from a measure which was designed to be unidimensional [16]. Analysis using the Rasch model demonstrated that the measure was unidimensional and that there was no DIF between the Dutch subgroups. DIF was detected between the UK and Dutch participants in two items (“getting food stuck in teeth” and “cried about my teeth”) when assessing those children with caries. This may be due to the differences in the two populations, as the UK children were younger (mean age = 8 years) and had higher levels of active caries (mean number of decayed teeth 0.36 in Dutch participants versus 6.01 in UK participants). This may have meant that two groups were more likely to answer these questions in a different way. Further analysis of this using the CARIES-QC-NL is required in a younger population with higher levels of active caries to confirm this finding.

The CARIES-QC-NL demonstrated weak but statistically significant correlations with the clinical data. These were slightly stronger when considering those only with caries, indicating acceptable construct validity. Weak correlations may be expected in a sample with relatively low levels of active dental caries, as the CARIES-QC is specifically designed to be used to measure changes in OHRQoL following interventions for dental caries. Interestingly in the overall sample, slightly higher correlations were found between the CARIES-QC-NL total score and missing and filled teeth, than with decayed teeth. This may be related to the small number of children who had untreated caries but does indicate that there may be continuing impacts from the treatment of dental caries. In the original UK validation study, it was found that not all children reported an improvement in their OHRQoL following treatment (*n* = 9; 26%) and indeed, the majority of children still reported one or more impacts following treatment (*n* = 28; 82%) [16]. A Brazilian study assessing the impact of untreated and treated caries found that children with teeth that were missing as a result of caries had Child Perceptions Questionnaire (11–14) scores 24% higher than those without caries experience [22]. An increase in impacts on OHRQoL resulting from the number of missing teeth has also been found in Brazilian adults [23,24].

The CHU9D has previously been used in the assessment of oral health impacts [25]. Foster Page and colleagues found that although the CHU9D was found to respond in the appropriate direction, it was not statistically significant, which indicates that it may be less sensitive to changes in oral health when compared to a measure of OHRQoL. In the present study, a relationship was demonstrated between the CARIES-QC-NL and CHU9D-NL, suggesting that there are changes in general wellbeing associated with impacts on oral health. The CHU9D mean values were 0.93 in the current study compared with 0.87 in the New Zealand study. The higher values found in this study may relate to the fact that the New Zealand population had a significantly higher DMFT (2.4 compared with 0.71), therefore, the children in the present study may have had fewer impacts on their wellbeing. In addition, the New Zealand study used the UK value set, whereas this study used the Dutch valuation set. Further studies using this Dutch valuation are required to put these results in context. Nonetheless, the correlation between the CARIES-QC-NL and CHU9D-NL identified by this study is important and provides evidence in support of the hypothesized association between OHRQoL and HRQoL. However, as the CHU9D-NL had limited correlations with clinical data, it may be that this is an indirect effect and further research is required to elicit this relationship.

To the authors’ knowledge, CHU9D is the only generic preference-based measure of health-related quality of life that has been used in studies of dental caries in children. Nonetheless, the present study found no statistically significant correlation between the CHU9D-NL and the clinical variables, nor was there any notable difference between the utility scores for children with and without caries. This fits with the issues identified in the aforementioned study by Foster Page and co-workers [25]. Despite a lack of research in this area, it could be assumed that other generic preference-based measures would suffer similar, if not further limitations, particularly since children were not involved in their development. These limitations can cause issues for researchers in quantifying the benefits of dental interventions in terms of QALYs. The use of QALYs offers multiple benefits to researchers and decision-makers alike, primarily through allowing direct comparison of interventions across different fields, without the complications of varied outcome measures being used; a common hindrance to cost-effectiveness analyses. It is possible that the limitations of the CHU9D and other generic measures could be overcome by using a preference-based measure specific to dental caries, though there is not currently such a measure in existence.

There are some limitations to this study, which should be considered. The first of these is the use of CARIES-QC-NL in this population. The CARIES-QC is designed to evaluate the change in OHRQoL following an intervention for dental caries, it is not designed to discriminate between groups. Indeed, the mean scores between the group with caries-experience and those without were not significantly different, although the mean score was slightly higher in the group with caries-experience. The CARIES-QC-NL was found to be valid and reliable in this sample, however, further studies are required to confirm its responsiveness in a Dutch population. Secondly, the Dutch valuation set for the CHU9D has not previously been used in a study such as this, therefore, it is difficult to draw comparisons with other studies, which have used different valuation systems. Finally, the study population was limited to four cities in the Netherlands, where children had fairly low levels of dental caries and the majority came from high socioeconomic status households.

In conclusion, this study demonstrated the validity and reliability of the CARIES-QC-NL and found a significant relationship between OHRQoL and generic wellbeing. Further evaluation of these measures in a Dutch population is required to demonstrate their utility in detecting change following interventions for untreated dental caries.

## Figures and Tables

**Table 1:** Demographic characteristics of sample of completed CARIES-QC questionnaires.

Variable	Proportion	Number
Gender		
Male	48.8%	237
Female	51.2%	249
Ethnicity		
Born in The Netherlands	90.2%	438
Born in the Netherlands but speak additional language at home	4.6%	22
Born outside Netherlands background	4.6%	22
Missing	0.8%	4
Socioeconomic status		
Lower	4.9	24
Middle	32.7	159
Higher	62.4	303

**Table 2:** Caries experience of the sample.

Caries-related Variables	Minimum	Maximum	Mean (SD)
D_3_MFT	0	8	0.71 (1.2)
Number of carious teeth	0	4	0.36 (0.75)
Number of missing teeth	0	2	0.1 (0.12)
Number of filled teeth	0	7	0.34 (0.85)
D_3_MFS	0	11	0.81 (1.4)
Number of carious surfaces	0	4	0.37 (0.77)
Number of missing surfaces	0	4	0.02 (0.27)
Number of filled surfaces	0	9	0.41 (1.1)

**Table 3:** Percentage of distribution of CARIES-QC in children with and without caries experience.

Item and Responses	Caries Group ICDAS ≥ 4 (*n* = 184)	Caries-Free Group ICDAS 0–3 (*n* = 302)	X^2^	*p*
1: Teeth hurt			8.85	**0.01**
not at all	80%	88%		
a bit	19%	11%		
a lot	1%	1%		
2: Hard to eat some foods			6.49	**0.04**
not at all	86%	91%		
a bit	14%	9%		
a lot	0	0		
3: Eat on one side of the mouth			2.58	0.28
not at all	87%	91%		
a bit	10%	8%		
a lot	3%	1%		
4: Food get stuck in my teeth			6.46	**0.04**
not at all	76%	85%		
a bit	23%	15%		
a lot	1%	0		
5: Teeth kept me awake			2.89	0.24
not at all	96%	98%		
a bit	3%	2%		
a lot	1%	0		
6: Teeth annoy me			3.57	0.17
not at all	84%	89%		
a bit	14%	11%		
a lot	2%	0		
7: Teeth hurt while brushing			1.21	0.54
not at all	85%	88%		
a bit	14%	11%		
a lot	1%	1%		
8: Eating more carefully			1.53	0.47
not at all	83%	86%		
a bit	16%	13%		
a lot	1%	1%		
9: Eating more slowly			1.57	0.46
not at all	90%	93%		
a bit	9%	4%		
a lot	1%	1%		
10: Feeling cross because of teeth			2.74	0.25
not at all	89%	93%		
a bit	8%	6%		
a lot	3%	1%		
11: Cried because of teeth			1.01	0.59
not at all	92%	94%		
a bit	7%	5%		
a lot	1%	1%		
12: Difficult to do schoolwork			1.33	0.51
not at all	97%	98%		
a bit	3%	2%		
a lot	0	0		
Global question			3.17	0.21
not at all	61%	66%		
a bit	29%	22%		
a lot	10%	11%		

**Table 4:** CARIES-QC data by subgroup.

Participants/Subgroups	Number (%)	Mean Score	CARIES-QC Score			
			Min	Max	Range	SD
Overall	486	13.29	12	28	16	2.53
Gender						
Female	251 (52%)	13.04	12	24	12	2.11
Male	235 (48%)	13.56	12	28	16	2.89
Ethnicity						
Indigenous	438 (90%)	13.20	12	28	16	2.51
Not indigenous	48 (10%)	14.21	12	22	10	2.66
Deprivation						
Deprivation group 1 (lowest)	24 (5%)	15.61	12	25	13	3.82
Deprivation group 2 (middle)	159 (33%)	13.39	12	28	16	2.74
Deprivation group 3 (highest)	303 (63%)	13.07	12	24	12	2.21
Group						
Caries-free	302 (61%)	13.19	12	25	0–13	2.49
Caries experience	184 (39%)	13.49	12	28	0–16	2.61

**Table 5:** Bivariate correlations between CARIES-QC-NL total scores and CHU9D-NL utility scores in ICDAS 0–3 and ICDAS ≥ 4 groups.

	CHU9D-NL Child	CHU9D-NL Proxy
**ICDAS 0–3 (*n* = 302)**		
CARIES-QC-NL	*r* = 0.11 (*p* = 0.05)	*r* = 0.084 (*p* = 0.13)
CHU9D-NL child	-	*r* = 0.183 (*p* = 0.01)
**ICDAS ≥ 4 (*n* = 184)**		
CARIES-QC-NL	*r* = 0.032 (*p* = 0.64)	*r* = 0.038 (*p* = 0.59)
CHU9D-NL child	-	*r* = 0.156 (*p* = 0.02)
**Total (*n* = 386)**		
CARIES-QC-NL	*r* = 0.066 (*p* = 0.13)	*r* = 0.30 (*p* = −0.48)
CHU9D-NL child		*r* = 0.19 (*p* < 0.001)

## Supplementary Materials

CARIES-QC is available on request at https://www.sheffield.ac.uk/dentalschool/research/create/home2#tab01.
